# Posterior Malleolus Fixation in Trimalleolar Ankle Fractures: Outcomes From a Single-Centre Retrospective Cohort

**DOI:** 10.7759/cureus.101072

**Published:** 2026-01-08

**Authors:** Smit Shah, André Fernandes, Fleur Shiers-Gelalis, Rohan Bidwai, Ilhan Alcelik, Mohamed Mahmoud, Vishal Upadhyay, James Stanley, Adam Budgen, Charlie Jowett

**Affiliations:** 1 Trauma and Orthopaedics, York and Scarborough Teaching Hospitals NHS Foundation Trust, York, GBR; 2 Trauma and Orthopaedics, Calderdale and Huddersfield NHS Foundation Trust, York, GBR; 3 Trauma and Orthopaedics, Queen Elizabeth Hospital Birmingham, Birmingham, GBR; 4 Trauma and Orthopaedics, York University Hospital, York, GBR; 5 Orthopaedics, York and Scarborough Teaching Hospitals NHS Foundation Trust, York, GBR

**Keywords:** ankle fractures, olerud-molander ankle score (omas), posterior malleolar fractures, posterior malleolus fixation, trimalleolar ankle fracture

## Abstract

Background: Fixation of the posterior malleolus in ankle fractures has evolved from simple size-based criteria to morphology-guided indications informed by computed tomography (CT). Direct posterior approaches now permit anatomic reduction of the posterior tibial plafond and restoration of syndesmotic stability. However, real-world functional outcomes and complication data from United Kingdom (UK) centres remain limited.

Objective: To describe patient-reported outcomes and complication patterns following morphology-guided posterior malleolus fixation for trimalleolar ankle fractures in a single National Health Service (NHS) teaching hospital.

Methods: We conducted a retrospective cohort study of patients with trimalleolar ankle fractures who underwent posterior malleolus fixation between 2020 and 2022. Functional outcome was assessed using the Olerud-Molander Ankle Score (OMAS). Scores were categorised as poor (<30), fair (30-59), good (60-79), very good (80-99), and excellent (≥100). Complications and reoperations were identified from electronic records. All summary data are reported as frequency (n) and percentage (%), and figures were generated directly from the clinical dataset.

Results: Thirty-nine patients contributed OMAS data. The median OMAS was 85, with an interquartile range of 27.5, indicating a distribution skewed towards higher function. Category distribution was poor in 2/39 (5.1%) patients, fair in 6/39 (15.4%), good in 8/39 (20.5%), very good in 16/39 (41.0%), and excellent in 7/39 (17.9%). Minor complications occurred in 10/39 patients (25.6%), most commonly pain in 2/39 (5.1%), post-traumatic arthritis requiring steroid injection in 2/39 (5.1%), and issues related to syndesmotic screws in 2/39 (5.1%). Seven patients (17.9%) underwent removal of metalwork. No deep infections or permanent neurological deficits were recorded.

Conclusion: Morphology-guided posterior malleolus fixation in trimalleolar ankle fractures was associated with high functional scores and an acceptable complication profile in this single-centre series. These findings support contemporary practice that emphasises CT-based assessment and direct posterior fixation when morphology or instability indicates, while highlighting the need for further prospective studies with standardised reporting and long-term follow-up.

## Introduction

Posterior malleolar fractures are a clinically important component of ankle injuries because of their impact on articular congruity, load transmission across the tibial plafond, and integrity of the distal tibiofibular syndesmosis. The posterior fragment provides the attachment site for the posterior inferior tibiofibular ligament (PITFL), and disruption or malreduction may therefore compromise syndesmotic stability and ankle function [1-3]. Historically, the decision to fix the posterior malleolus was guided largely by fragment size, with thresholds of 25-33% of the tibial plafond widely cited as indications for operative fixation. Subsequent experience and the advent of routine CT scanning have shown that fragment size alone is an unreliable surrogate for instability or incongruity [1-3].

Modern CT allows detailed assessment of posterior fragment morphology, including posterolateral obliquity, posteromedial extension, degree of incisural involvement, and comminution. Haraguchi et al. first described morphologic patterns that correlate more closely with instability than simple fragment percentage [1]. Bartoníček and colleagues later proposed classification systems that integrate these morphologic features and guide the choice of surgical approach [3]. These developments have contributed to a paradigm shift in which morphology and syndesmotic stability, rather than size alone, are viewed as the primary determinants of whether to fix the posterior malleolus.

Parallel to this conceptual evolution, surgical techniques for posterior malleolus fixation have advanced. Direct posterior approaches, most commonly the posterolateral and posteromedial approaches, permit visualised reduction of the posterior plafond and incision, as well as application of posterior-to-anterior lag screws or buttress plates. Biomechanical studies have shown that fixation of the posterior fragment can restore PITFL tension and improve syndesmotic stability to a degree comparable with or superior to trans-syndesmotic screw fixation [4,5]. Clinical series from specialist centres report high rates of anatomic reduction, favourable functional outcomes, and low rates of malreduction when the posterior component is addressed directly [2,6].

Despite increasing adoption of morphology-guided posterior fixation, real-world data from UK NHS settings remain scarce. Variability in follow-up schedules, inconsistent capture of patient-reported outcome measures (PROMs), and heterogeneity in operative techniques can all limit benchmarking against published series. The Olerud-Molander Ankle Score (OMAS) is a pragmatic PROM that summarises patient perception of pain, stiffness, swelling, functional activities, and return to work into a single 0-100 score [1]. It is widely used in ankle fracture research and provides a useful tool for comparing cohorts.

The aim of this study is to describe functional outcomes and complications following posterior malleolus fixation for trimalleolar ankle fractures at a single NHS teaching hospital. We report OMAS outcomes, complication patterns, and reoperations based on real clinical data, and we present figures derived directly from the dataset. Importantly, we avoid reliance on synthetic or illustrative figures, in line with journal guidance, and focus on morphology-guided practice reflecting a contemporary NHS setting.

## Materials and methods

We conducted a retrospective observational study at an NHS teaching hospital, UK. Patients with trimalleolar ankle fractures who underwent fixation of the posterior malleolus were identified from operative databases and outpatient clinic records between November 2020 and October 2022. The decision to fix the posterior fragment was made at the discretion of the treating surgeon, guided by CT-defined fracture morphology and intraoperative stability assessment. Ethical approval was obtained, and all data were anonymised prior to analysis.

During the study period, 146 patients sustained trimalleolar ankle fractures, of whom 76 underwent posterior malleolus fixation. From this operative cohort, 39 patients met the inclusion criteria and consented to longitudinal functional follow-up using the Olerud-Molander Ankle Score (OMAS). Inclusion criteria were age ≥16 years, CT-confirmed trimalleolar fracture with posterior malleolus fixation, and availability of at least one postoperative OMAS assessment beyond 12 months. Patients were excluded if they did not undergo posterior fixation, had incomplete or missing functional outcome data, or had polytrauma or neurological conditions precluding reliable PROM assessment. These 39 patients were followed at standard postoperative intervals (6 weeks, 12 weeks, 6 months, 1 year, and 24 months), with OMAS recorded at each review. Actual attendance varied among individuals, reflecting real-world clinical practice, and all available OMAS scores up to 24 months were included in the analysis to provide an authentic representation of functional outcomes in routine NHS care.

Surgical strategy

The decision to fix the posterior malleolus was based on CT-defined morphology and intraoperative assessment of stability rather than fragment size alone. Indications included posterolateral or posteromedial fragments with incisural extension, comminuted posterior fragments associated with plafond incongruity, evidence of syndesmotic instability on stress testing, and inferred PITFL disruption from CT or direct visualisation. Surgical approach selection followed the Liverpool algorithm described by Mason and Molloy, which links fracture morphology to the preferred operative approach and fixation strategy [6] (Table 1).

**Table 1:** Liverpool algorithm for direct fixation of the posterior malleolus (Mason & Molloy).

Classification	Treatment	Approach
1	Syndesmotic Fixation	Direct lateral (fibular)
2A	ORIF	Postero-lateral (PL)
2B	ORIF Postero-medial Fragment First	PL + MPM (Medial Posterior-medial) or PM
3	ORIF	Posterior-medial (PM)

This algorithm was used to guide operative planning in all cases. Posterolateral or posteromedial approaches were selected in accordance with fragment morphology and stability requirements [6]. Fixation techniques included posterior-to-anterior lag screws, buttress plating, or combined constructs as indicated. Concomitant fibular fixation and syndesmotic stabilisation were performed when required.

Outcome measures

The primary outcome was the OMAS at the latest documented follow-up. OMAS ranges from 0 (worst function) to 100 (best function) and includes domains of pain, stiffness, swelling, stair-climbing, running, jumping, squatting, supports, and work capacity. For interpretability, OMAS values were grouped into standard categories, defined as poor (<30), fair (30-59), good (60-79), very good (80-99), and excellent (≥100). Secondary outcomes were complications and reoperations. Complications were categorised as wound problems, pain, stiffness, syndesmotic screw-related issues, post-traumatic arthritis requiring injection, and metalwork irritation. Reoperations included removal of metalwork, removal of syndesmotic screws, and steroid injections for post-traumatic arthritis.

Data collection and analysis

Demographic data, operative details, complications, and reoperations were retrieved from electronic patient records. OMAS scores were collected during outpatient clinic visits or via structured telephone review using the original OMAS questionnaire. For analysis, the latest available OMAS per patient was used.

Data are summarised using medians and interquartile ranges for continuous variables and frequency (n) with percentage (%) for categorical variables. OMAS distributions were illustrated with a boxplot and histogram, and complications were displayed as a bar chart.

## Results

Cohort description

A total of 39 patients met inclusion criteria and had complete OMAS follow-up. All sustained trimalleolar ankle fractures were treated with posterior malleolus fixation as part of operative management. Demographic details, specific implant choices, and operative approaches were heterogeneous, reflecting real-world practice within a trauma service.

Olerud-Molander ankle score

**Figure 1:**
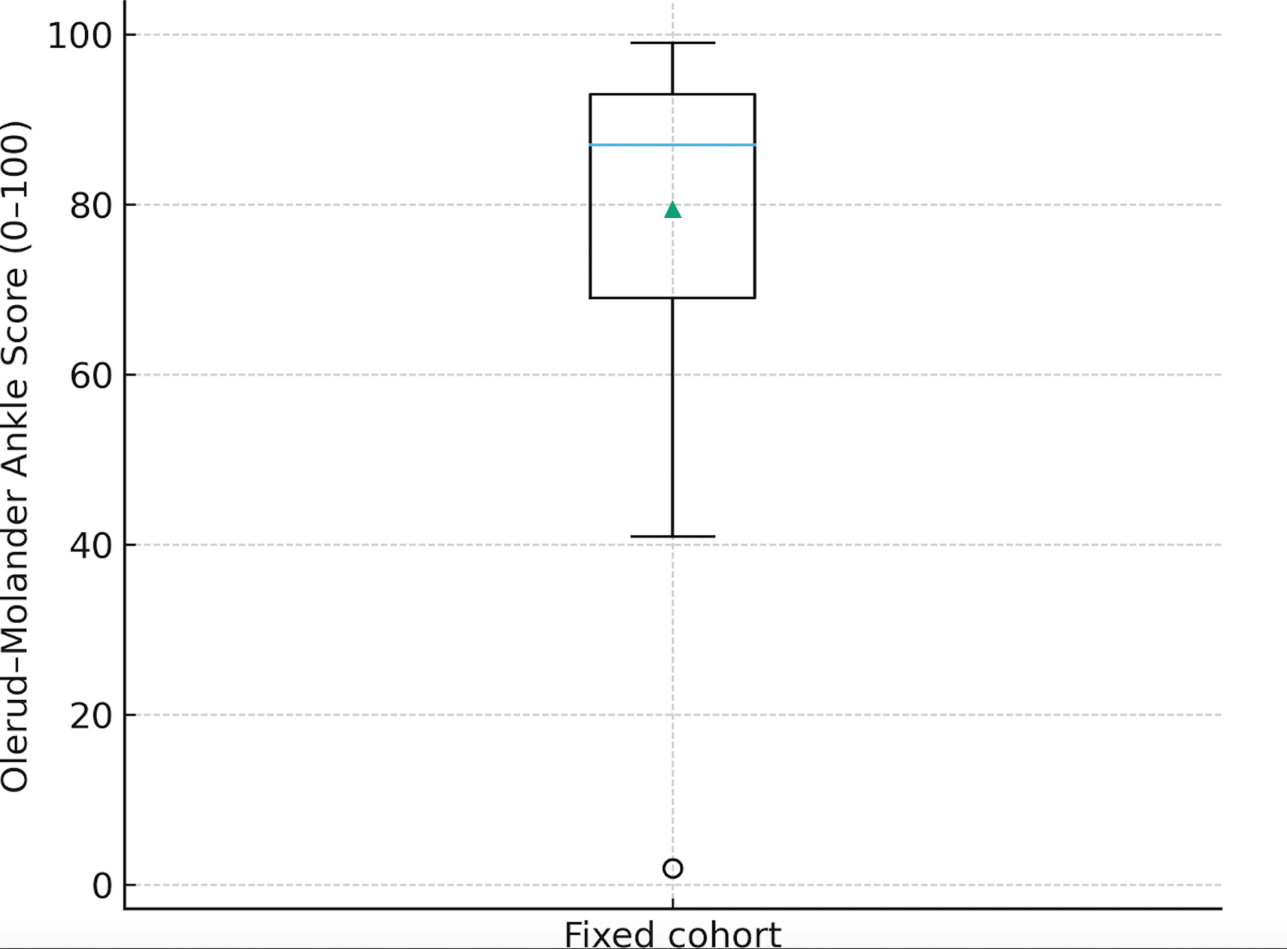
Boxplot illustrating the distribution of Olerud–Molander Ankle Scores (OMAS) for the cohort.

**Figure 2:**
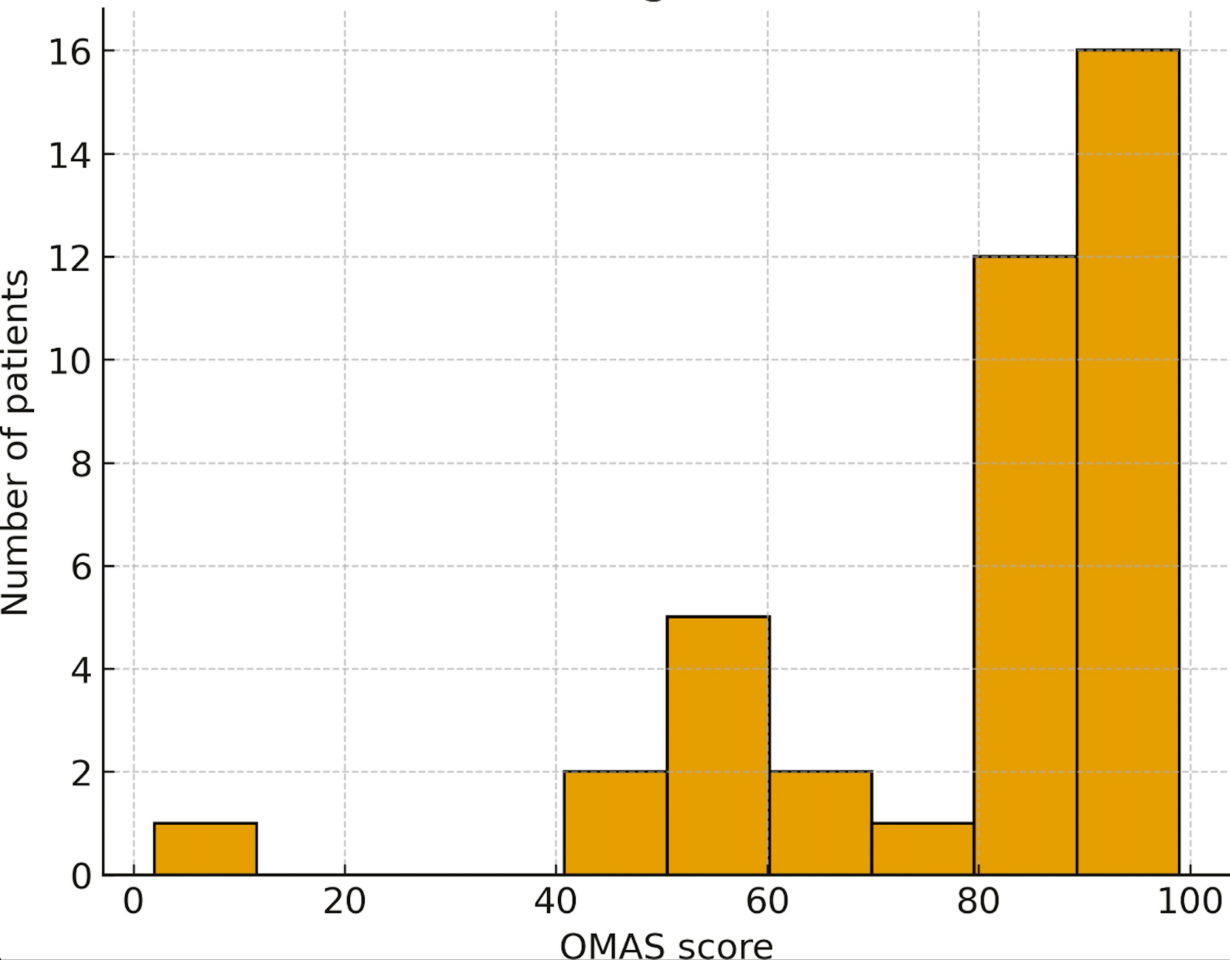
Histogram showing the frequency distribution of Olerud–Molander Ankle Scores (OMAS).

OMAS values across the cohort ranged from 15 to 105. The median OMAS was 85, with an interquartile range of 27.5, indicating a distribution skewed towards the higher end of the scale. The category distribution was poor in 2/39 (5.1%) patients, fair in 6/39 (15.4%), good in 8/39 (20.5%), very good in 16/39 (41.0%), and excellent in 7/39 (17.9%). Thus, 23/39 patients (59.0%) achieved very good or excellent scores, and only 2/39 (5.1%) remained in the poor category at the latest follow-up. Figure 1 (boxplot) and Figure 2 (histogram) illustrate the distribution of OMAS scores.

Complications

**Figure 3:**
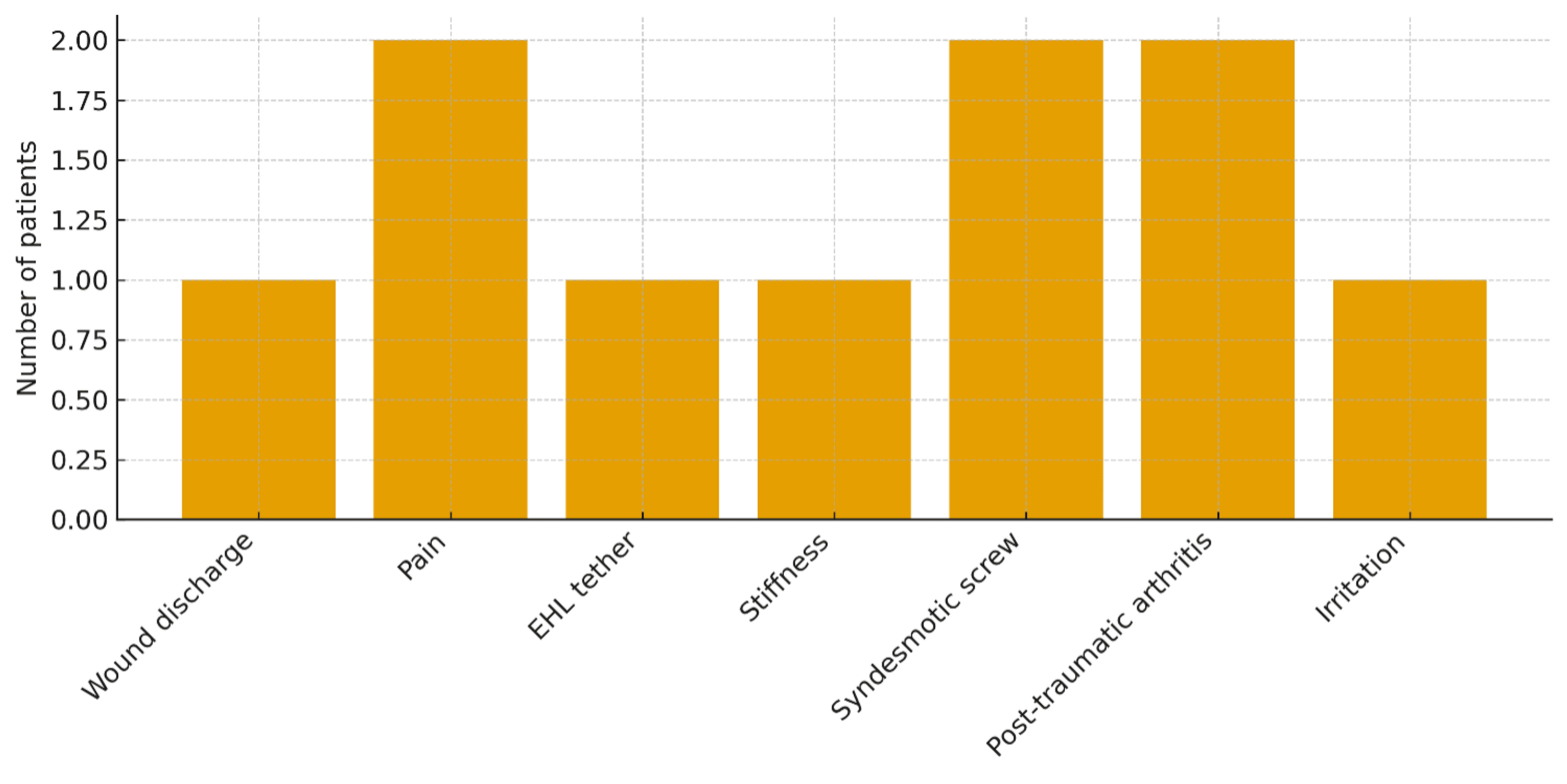
Bar chart summarising the distribution of recorded complications following posterior malleolus fixation.

Complications were recorded in 10/39 patients (25.6%). These consisted of wound discharge in 1/39 (2.6%) patients, pain as a primary complaint leading to further assessment or metalwork removal in 2/39 (5.1%), extensor hallucis longus tethering associated with posterior hardware in 1/39 (2.6%), stiffness requiring targeted physiotherapy or intervention in 1/39 (2.6%), problems attributed to syndesmotic screws such as irritation, screw breakage, or planned removal for symptoms in 2/39 (5.1%), post-traumatic arthritis treated with local anaesthetic and steroid injection in 2/39 (5.1%), and metalwork irritation necessitating removal in 1/39 (2.6%). No deep infections, non-union of the posterior fragment or permanent neurological deficits were documented. The distribution of complication types is summarised in Figure 3.

Reoperations

Seven patients (17.9%) underwent removal of metalwork, usually combined with symptomatic relief of irritation or stiffness. Two patients (5.1%) received intra-articular steroid injections for symptomatic post-traumatic arthritis. Several patients had planned removal of syndesmotic screws, often in conjunction with the above procedures. Reoperations were therefore relatively common but were largely elective or planned and did not typically represent failures of fixation.

## Discussion

This single-centre retrospective cohort study demonstrates that morphology-guided posterior malleolus fixation in trimalleolar ankle fractures can achieve favourable functional outcomes with an acceptable complication profile in an NHS trauma setting. The majority of patients achieved very good or excellent OMAS scores at the latest follow-up, and only a small proportion reported poor function. These findings align with contemporary literature and support the ongoing shift from size-based to morphology-based indications for posterior fixation.

The historical practice of relying on fragment size as the sole operative threshold has been challenged by multiple authors [2,3,7-10]. CT-based studies have demonstrated that posterolateral fragment orientation, commissural extension, and involvement of the fibular incisura are more directly related to syndesmotic instability and articular incongruity than the percentage of plafond involvement alone [2,3,9-12]. Our cohort selection reflected this modern philosophy, with fixation performed when morphology suggested potential instability or loss of posterior buttress, rather than on the basis of size thresholds.

Biomechanical work underpins this approach. Gardner et al. and Miller et al. have shown that fixation of the posterior malleolus restores PITFL tension and improves syndesmotic stability, sometimes obviating the need for trans-syndesmotic screw fixation [4,5,13-15]. Clinical series such as those by Mason and Verhage have reported that direct posterior fixation is associated with improved reduction quality and lower rates of malreduction [6-9,11]. The high proportion of Very Good and Excellent OMAS scores in our study is consistent with these reports and suggests that morphology-guided posterior fixation contributes meaningfully to functional recovery.

Complications in this series were relatively infrequent and predominantly minor. Wound issues occurred in a small minority, and there were no deep infections. Complications associated with syndesmotic screws and post-traumatic arthritis were managed with screw removal or injection, respectively. These findings are broadly in keeping with other reports of posterior approaches, which emphasise careful soft-tissue handling, attention to the sural nerve, and meticulous closure to minimise wound problems [6,8,16]. The complication bar chart (Figure 3) illustrates that no single complication type dominated, underscoring the overall safety of the approach when performed in an experienced setting.

Reoperations were mostly planned or elective, including removal of prominent hardware or symptomatic syndesmotic screws and injections for arthritis. This pattern is typical of ankle fracture practice and should be interpreted in the context of shared decision-making and patient expectations rather than as a direct measure of surgical failure [7,8]. From a patient-centred perspective, the important observation is that reoperations were seldom associated with catastrophic complications such as deep infection or non-union.

Strengths of this study include the use of a validated PROM (OMAS), reliance on real clinical data rather than synthetic distributions, and adherence to explicit morphology-based operative criteria within a contemporary NHS setting [9]. The figures presented are directly derived from the dataset and therefore accurately represent outcome distributions and complication patterns.

Several limitations must be acknowledged. The retrospective design introduces the possibility of selection bias and incomplete documentation. The sample size is modest and limits subgroup analyses, for example, comparing fixation strategies or fragment morphologies. Follow-up intervals were not strictly standardised; OMAS scores were taken at the latest available assessment, which may vary between patients. In addition, radiographic measures such as articular step-off, tibiofibular clear space, or progression to osteoarthritis were not systematically collected and therefore cannot be correlated with functional outcomes.

Nonetheless, the findings contribute real-world evidence supporting morphology-guided posterior malleolus fixation. They highlight the feasibility of implementing CT-based algorithms and direct posterior approaches within an NHS trauma service and provide benchmark functional outcomes for similar centres.

Future research should include multicentre prospective studies with larger cohorts and standardised imaging protocols, such as CT-based classification using Haraguchi or Bartoníček systems [1,3]. Integration of long-term radiographic follow-up and additional PROMs, including generic measures such as EQ-5D or PROMIS, would further enrich understanding of patient recovery. Comparative studies evaluating posterior fixation versus indirect reduction or non-fixation in specific morphologic subgroups would also be valuable [10-13].

## Conclusions

Morphology-guided posterior malleolus fixation for trimalleolar ankle fractures was associated with favourable patient-reported functional outcomes and an acceptable complication profile in this single NHS teaching hospital cohort. In practice, direct posterior fixation restores the posterior tibial buttress, re-tensions the PITFL, and improves incisural congruency, mechanical prerequisites for durable ankle function. These findings support contemporary practice that prioritises CT-based morphology and stability assessment as an aid in decision-making. Further adequately powered prospective studies with standardised CT morphology reporting, rigorous PROM capture, and long-term radiographic follow-up, including post-traumatic osteoarthritis, are warranted. 
